# Secreted Phospholipase A_2_-IIA Modulates Transdifferentiation of Cardiac Fibroblast through EGFR Transactivation: An Inflammation–Fibrosis Link

**DOI:** 10.3390/cells9020396

**Published:** 2020-02-08

**Authors:** Ruben Martin, Beatriz Gutierrez, Claudia Cordova, Alberto San Roman, Yolanda Alvarez, Marita Hernandez, Victoria Cachofeiro, Maria L Nieto

**Affiliations:** 1 Instituto de Ciencias del Corazón, Hospital Clínico Universitario, 47003 Valladolid, Spain; rubenpmm@hotmail.com (R.M.); asanroman@secardiologia.es (A.S.R.); 2 Instituto de Biología y Genética Molecular, CSIC-Universidad de Valladolid, 47003 Valladolid, Spain; bgutimiranda@gmail.com (B.G.); cmpcmarcos@gmail.com (C.C.); yalvarez@ibgm.uva.es (Y.A.); maritahg@ibgm.uva.es (M.H.); 3 CIBER de Enfermedades Cardiovasculares (CIBERCV). Instituto de Salud Carlos III, - 28029 Madrid, Spain; 4 Departamento de Fisiología, Facultad de Medicina, Universidad Complutense de Madrid, Spain. Instituto de Investigación Sanitaria Gregorio Marañón (IiSGM), 28040 Madrid, Spain; vcara@ucm.es

**Keywords:** cardiac fibroblast, epidermal growth factor receptor, lysyl oxidase, fibrosis, myocarditis, secreted phospholipase A_2_

## Abstract

Secreted phospholipase A_2_-IIA (sPLA_2_-IIA) is a pro-inflammatory protein associated with cardiovascular disorders, whose functions and underlying mechanisms in cardiac remodelling are still under investigation. We herein study the role of sPLA_2_-IIA in cardiac fibroblast (CFs)-to-myofibroblast differentiation and fibrosis, two major features involved in cardiac remodelling, and also explore potential mechanisms involved. In a mice model of dilated cardiomyopathy (DCM) after autoimmune myocarditis, serum and cardiac sPLA_2_-IIA protein expression were found to be increased, together with elevated cardiac levels of the cross-linking enzyme lysyl oxidase (LOX) and reactive oxygen species (ROS) accumulation. Exogenous sPLA_2_-IIA treatment induced proliferation and differentiation of adult rat CFs. Molecular studies demonstrated that sPLA_2_-IIA promoted Src phosphorylation, shedding of the membrane-anchored heparin-binding EGF-like growth factor (HB-EGF) ectodomain and EGFR phosphorylation, which triggered phosphorylation of ERK, P70S6K and rS6. This was also accompanied by an up-regulated expression of the bone morphogenic protein (BMP)-1, LOX and collagen I. ROS accumulation were also found to be increased in sPLA_2_-IIA-treated CFs. The presence of inhibitors of the Src/ADAMs-dependent HB-EGF shedding/EGFR pathway abolished the CF phenotype induced by sPLA_2_-IIA. In conclusion, sPLA_2_-IIA may promote myofibroblast differentiation through its ability to modulate EGFR transactivation and signalling as key mechanisms that underlie its biological and pro-fibrotic effects.

## 1. Introduction

Inflammation and autoimmunity are involved in the progression of many heart diseases. Myocarditis is a precursor of dilated cardiomyopathy (DCM) and represents the most common cause of chronic heart failure or even sudden death; nevertheless, little is known about the mechanisms involved in post-inflammatory cardiac remodelling leading to DCM [1,2,3]. Significant steps in the progression from myocarditis to DCM and heart failure involve extracellular matrix (ECM) remodelling and fibrosis, which is characterised by the abundant production of ECM proteins resulting in a change of the structure and architecture of the myocardium, thus impairing the ventricular contractility and functionality [4,5]. The fact that researchers have found that components released during acute myocarditis drive changes in heart structure, which appear several weeks or months later, deepen the characterisation of these key pathogenic factors as having important clinical relevance [2,3,4]. In experimental autoimmune myocarditis (EAM), a mouse model of myocarditis, it has been demonstrated that overproduction of pro-inflammatory proteins such as TNFα, TGFβ and IL-1β play important roles by affecting the production of ECM proteins and ECM-regulatory proteins [6,7,8].

In the physiological turnover of the ECM, as well as its pathological remodelling, cardiac fibroblasts (CFs) play a central role [9]. In response to myocardial injury, CFs undergo three important phenotypic changes: (1) they differentiate into myofibroblast, which are more mobile and contractile, (2) they proliferate, and (3) they produce ECM components, which in turn may have an impact on their functions [10,11]. Among the ECM macromolecules produced by activated CFs, periostin, bone morphogenetic protein-1 (BMP-1) and lysyl oxidase (LOX) play a key role in promoting collagen cross-linking, which determined mechanical properties of the ECM, thus making it less prone to degradation [12,13].

Secretory phospholipase A_2_s (sPLA_2_s) constitute a family of up to 10 proteins, which play critical roles under various physiological and pathophysiological conditions. Each individual sPLA_2_ exhibits unique tissue and cellular distributions and enzymatic properties, suggesting their distinct biological roles.

Among sPLA_2_s, the sPLA_2_-IIA sub-family is known to be a determining factor in the pathogenesis of a wide range of inflammatory disorders [14]. Its plasma concentration markedly increases in diseases that involve systemic inflammation, such as sepsis, rheumatoid arthritis and cardiovascular disease (up to 1000-fold and >1 μg/mL). Related to pathologies affecting the heart and cardiovascular system, its presence is correlated with adverse vascular complications [15,16,17,18]. sPLA_2_-IIA has been detected in atherosclerotic lesions, these being considered an independent risk factor for coronary artery disease and a predictor for clinical coronary events. Proatherogenic actions of sPLA_2_-IIA involve lipoprotein remodelling due to its enzymatic actions, as well as induction of monocyte differentiation to dendritic cells and maturation due to its cytokine-like activity [19]. Additionally, approaches using mice transfected with the human *sPLA*_2_*–IIA* gene have evidenced increased collagen in atherosclerotic lesions [20]. Moreover, it has been shown that the treatment of spontaneously hypertensive rats with an sPLA_2_-IIA inhibitor prevents cardiac fibrosis [21].

In infarcted hearts, expression of sPLA_2_-IIA was markedly increased in damaged cardiomyocytes, and it has been associated with the ischemia-related death of cardiac myocyte [22,23]. Despite all this evidence, it remains a challenge to understand the effects and the signalling pathways that sPLA_2_-IIA may trigger in cardiac fibroblasts, as well as their role in the pathological remodelling and fibrosis in the heart.

## 2. Materials and Methods

### 2.1. Materials

A C127 mouse fibroblast cell line stably transfected with the coding sequence of sPLA_2_-IIA from human placenta was kindly provided by Dr. Olivier and used as a source of human recombinant enzyme, and it was obtained and purified as described previously [24]. Rapamycin and other chemicals were from Sigma Chemical Co. PD98059 and AG1478 inhibitors were from Tocris Biosciece. Hybond-P membrane was from Amersham Biosciences.

### 2.2. Animals and Immunization

BALB/c mice from Charles River Laboratories were housed in the animal care facility at the Medical School of the University of Valladolid (UVa) and were provided food and water ad lib, under standard conditions. All experimental protocols were reviewed and approved by the Animal Ethics Committee of the UVa (Project number 6203828) and were in accordance with European legislation (86/609/EU).

Disease was induced in 6–8 week-old male mice by immunisation at day 0 with 50 μg of the murine specific α-myosin-heavy chain-derived acetylated peptide (MyHCα_614–629_), as was previously described [25]. MyHCα_614–629_ was generated in the peptide synthesis laboratory of Dr. F. Barahona (CBM, Madrid, Spain). After terminal anesthesia with xylazine/ketamine, mice were sacrificed either on day 21 or 65. The heart was removed and weighed.

### 2.3. Histological and Immunohistochemical Studies

Hearts were obtained on day 65 from control and EAM mice. One-half was fixed in 4% paraformaldehyde and embedded in paraffin and the other half was frozen at −80 °C. Embedded tissues were cut in 5 μm thick sections, stained with hematoxylin–eosin (H&E) and Masson’s trichrome (Sigma-Aldrich, St Louis, MO, USA), and examined by light microscopy. For the purposes of this study, each specimen was evaluated qualitatively with a Nikon Eclipse 90i microscope connected to a DS-Ri1 digital camera (Nikon Instruments Inc., Amstelveen, the Netherlands) with a 20× objective lens. Sections from 4–10 segments per mouse were examined blindly by two investigators. 

Immunohistochemistry was carried out on 5 µm sections mounted on lysine-coated glass. Tissue was permeabilized with Tween 20 for 15 min and blocked with 5% serum for 20 min at room temperature; antigen retrieval was by heat mediation in a citrate buffer. Samples were incubated with anti-LOX antibody (1/100 in 10% serum in TBS + 0.05% Tween) for 14 h at 4 °C. An FITC anti-rabbit IgG polyclonal (1/500) was used as the secondary antibody. Images were obtained on a Leica TCS SP5X confocal microscope (TCS Leica Microsystems, Mannheim, Germany). Bars 50 μm).

### 2.4. In Situ Detection of Superoxide Production

To evaluate in situ superoxide production from hearts, unfixed frozen 8 μm thick cross-sections were stained with 2 μM dihydroethidium (DHE; Molecular Probes, Eugene, OR, USA) at 37 °C for 30 min in a light-protected humidified chamber. Images were obtained with a Nikon Eclipse 90i inverted fluorescence microscope using 2× or 20× objective lenses. Red fluorescence was collected through a 590 nm filter after excitation of cells at 488 nm.

### 2.5. Measurements of sPLA_2_-IIA by an Enzyme-Linked Immunosorbent Assay (ELISA)

sPLA_2_-IIA levels were determined in both serum samples and heart tissue using a commercial ELISA (Cusabio Biotech Co, Wuhan, China), according to the manufacturer’s protocols. Heart tissue homogenates were prepared with the apical part of the heart, homogenised in 1 mL of ice-cold PBS, supplemented with a protease inhibitor cocktail (Sigma-Aldrich, St Louis, MO, USA). Samples were centrifuged at 800× *g* for 15 min at 4 °C. Total protein concentration in the supernatants was determined by using the Bradford method with bovine serum albumin (BSA) as standard. Data were processed and expressed as concentration of cytokine/mg of tissue or concentration of cytokine/mL for serum samples.

### 2.6. In Vitro Studies

#### 2.6.1. Cell Culture

Primary cultures of CFs were obtained from male Wistar rats weighing 250–300 g by differential centrifugation of cardiac cells released after mechanical and enzymatic digestion of the hearts [25]. Cells were grown on poly-L-lysine coated flasks in DMEM supplemented with 10% FCS, 10 mM l-glutamine, 100 U/mL penicillin/streptomycin, 10 mM pyruvate and 2 mM HEPES. These cells were labeled as P1 and used at 2–3 passages to minimise changes in phenotype associated with culture. None variation between individual fibroblast preparations was observed using routine phenotyping methods with anti-vimentin and anti-α-SMA antibodies.

#### 2.6.2. Cell Proliferation Assay

Cell proliferation was quantified by using the Promega kit, Cell Titer 96 ^®^Aqueous One Solution Cell Proliferation Assay, according to the manufacturer’s recommendations. Briefly, CFs were seeded on 96-well culture plates (20 × 10^3^ cells/well) and serum-starved for 24 h. Cells were pre-treated with either vehicle or the indicated inhibitor for 30 min before the addition of 1 μg/mL of sPLA_2_-IIA or 1 μM of angiotensin II (AngII). After 24 h of incubation, the proliferative response was quantified by recording the absorbance at 490 nm in a microplate reader (OD value). Formazan product formation is measured as an assessment of the number of metabolically active cells. Assays were each performed in quintuplicate, *n* = 3.

#### 2.6.3. Quantification of Collagen Deposition

In vitro fibrotic activity of sPLA_2_-IIA was assessed by picro-Sirius Red (pSR) staining and quantitative analysis, as previously was reported [26]. In brief, CFs cultured in 12-well plates were treated with 1 μg/mL of sPLA_2_-IIA at 37 °C for 72 h. Afterwards, cells were fixed in methanol overnight at 4 °C, carefully washed twice with PBS and incubated in the 0.1% pSR staining solution (Sigma-Aldrich) at room temperature for 1 h. The staining solution was removed and cells were washed three times with 0.1% acetic acid. Then, pSR was eluted in 0.1 N sodium hydroxide, 200 μL per well. Plates were placed on a rocking platform at room temperature for 1 h before determining the OD value at 540 nm with a VERSAmax Microplate Reader (Molecular Devices LLC, Sunnyvale, CA, USA). Experiments were performed in triplicate wells, *n* = 3.

#### 2.6.4. Measurement of ROS Production and Mitochondrial Inner Transmembrane Potential Detection

For detection of intracellular and mitochondrial ion superoxide (O_2_^−^) production, CFs were stimulated with 1 μg/mL of sPLA_2_-IIA at 37 °C, washed and then loaded with 2 µM of DHE for 30 min or with 4 μM of MitoSOX ^TM^ Red for 10 min, respectively, at 37 °C. For the detection of intracellular hydrogen peroxide (H_2_O_2_) production, CFs were preloaded with 10 µM of dichlorofluorescein diacetate (DCFH-DA) for 30 min at 37 °C, washed and then stimulated with 1 μg/mL of sPLA_2_-IIA. At the indicated times, fluorescent signals derived by reaction of DHE, MitoSOX^TM^ Red or DCF with ROS were analysed by recording FL3, FL2 and FL1 fluorescence, respectively, in a Gallius flow cytometer (Beckman Coulter, Brea, CA, USA). In some experiments, cells were pretreated for 30 min with the indicated inhibitor before incubation with the agonist. Experiments were repeated at least three times with similar results. The data are given as one representative histogram.

To evaluate mitochondrial transmembrane potential (Δψm), CFs were loaded with 4 μM rhodamine 123. After washing, cells were treated with 1 μg/mL of sPLA2-IIA for 6 or 24 h at 37 °C and analysed by flow cytometry.

#### 2.6.5. Flow Cytometric Analysis

CFs, 5 × 10^6^/flask, were treated with 1 μg/mL of sPLA_2_-IIA for the indicated times in the absence or presence of selected inhibitors at 37 °C. Cells to be analysed for expression of HB-EGF were fixed in a mixture of 4% paraformaldehyde in PBS for 15 min at room temperature before incubation with FITC-conjugated anti-HB-EGF antibody (Calbiochem, San Diego, CA, USA) for 1 h at 4 °C. For cellular LOX, BMP-1, paxillin, α-smooth muscle actin (α-SMA), TFGβ, phospho-EGFR and phospho-Src analysis, CFs were fixed in 4% paraformaldehyde for 15 min, washed with PBS and permeabilised with 0.3% Triton X-100 for 5 min. After that, cells were incubated with specific primary antibodies for 1 h at 4 °C, and then with a FITC-labelled secondary antibody for 45 min at 4 °C. After washing, the cells were analysed with a Flow Cytometer (Gallios^TM^; Beckman Coulter, Brea, CA, USA). Data analysis was performed using WinMDI 2.7 software.

#### 2.6.6. Measurements of LOX Enzymatic Activity

LOX enzyme activity was measured in the supernatant from CFs cultures by using the Amplite™ Fluorimetric Lysyl Oxidase Assay Kit (AAT Bioquest Inc., Sunnyvale, CA, USA), according to the manufacturer’s instructions. Parallel assays were prepared with 500 μM beta-aminopropionitrile fumarate (BAPN) to inhibit LOX activity and ensure assay specificity. Fluorescence was measured using a fluorescence spectrophotometer (Ex/Em = 535/590). Data are expressed as fold-change when compared with control conditions.

#### 2.6.7. Western Blot Analysis

After treatment, CFs were washed twice with PBS and harvested in Laemmli SDS sample buffer. Equal amounts of protein extracts were separated by SDS-PAGE and transferred to polyvinylidene difluoride membranes; after blocking, they were incubated for 18 h at 4 °C with primary antibodies against actin (Santa Cruz Biotechnology Inc, Santa Cruz, CA, USA), ERK 1/2 (Zymed Laboratories, San Francisco, CA, USA), p-ERK1/2 (Thr202/Tyr204), phospho-P70S6 kinase (p-P70S6K, Thr389), and phospho-S6 ribosomal protein (p-rS6, Ser235/236) (Cell Signaling Technology, Inc, Danvers, MA, USA). After washing, membranes were incubated with the corresponding secondary antibody (1:2000, *v/v*) conjugated with horseradish peroxidase at room temperature for 30 min. The blots were developed using enhanced chemiluminescence. Experiments shown here were repeated at least 3 times with reproducible results and a representative one is presented.

#### 2.6.8. Immunofluorescence Studies

Resting, AngII- and sPLA_2_-IIA-treated CFs, on 18-mm poly-l-lysine coated cover glass coverslips, were fixed in cold 4% paraformaldehyde for 10 min, permeabilised in 0.3% Triton X-100 for 10 min and blocked with 3% BSA for 1 h. Immunostaining with anti-α-SMA, LOX or BMP-1 antibodies (Abcam, Cambridge, UK) was performed at room temperature for 30 min, followed by incubation with FITC-conjugated secondary antibody. Cells were then analysed in a Nikon Eclipse 90i. Objective lens 20×.

### 2.7. Statistical Analyses 

Differences between 2 groups were analysed by unpaired Student’s *t*-test. Specific differences between more groups were analysed using 1-way ANOVA followed by Bonferroni’s test where appropriate. Results are described as mean ± SD. *p* < 0.05 was considered statistically significant. Statistical analyses were performed using the GraphPad Prism Version 4 software (San Diego, CA, USA).

## 3. Results

### 3.1. In Vivo Findings

#### sPLA_2_-IIA is Up-Regulated in MyCHα-Induced Myocarditis

**Figure 1:**
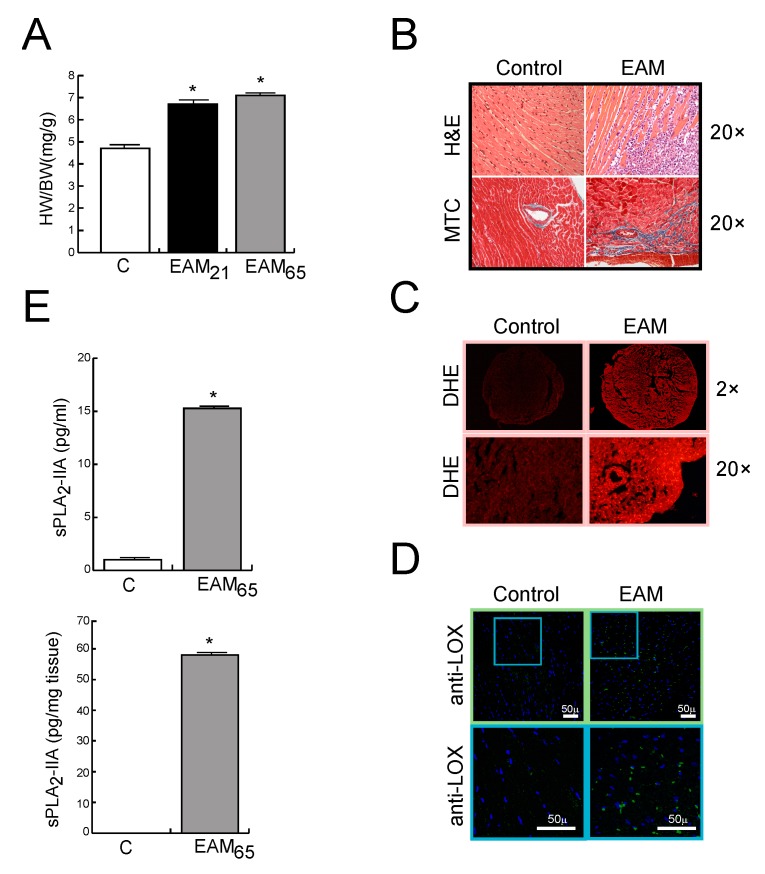
BALB/c mice were immunised with the MyCHα_614–629_ peptide. (**A**) Heart weight (HW)/body weight (BW) ratio of mice sacrificed 21 or 65 days after initial immunisation. (**B**–**D**) Representative photomicrographs of heart sections from control and EAM mice on day 65: (**B**) H&E and MTC stain, (**C**) DHE stain (for detection of O_2_^−^), and (**D**) LOX immunohistochemistry. Objective lens 2× and 20×, magnification 20× and 200×, respectively. (**C**) sPLA_2_-IIA protein levels on sera and heart tissue from control and EAM mice on day 65. Values are means ± SD. * *p* < 0.001 versus control mice, n = 7 in each group.

The expression of sPLA_2_-IIA was investigated in hearts from EAM-affected mice. MyCHα-immunised mice show an increase in HW/BW index of 40% and 51% higher, at days 21 and 65, respectively, compared to those of the healthy un-induced group (*p* < 0.001, Figure 1A). In addition, and confirming the myocarditis development, the histological analysis shows inflammatory infiltrates and fibrotic areas in the myocardium, evidenced by H&E and Masson’s trichrome staining, respectively, in EAM, but not in sham-immunised mice, at day 65 post-immunisation (Figure 1B). Moreover, and consistent with previously reported, ROS cardiac levels were higher in EAM mice than in control ones, as suggested by the stronger brightness (fluorescent intensity) (Figure 1C). Likewise, immunofluorescence staining of the key collagen cross-linking enzyme, LOX, showed a higher expression in the cardiac tissue of EAM mice than in control mice (Figure 1D). Figure 1E shows that the sPLA_2_-IIA protein was up-regulated in the hearts and serum from mice, while it was barely detectable in control mice.

### 3.2. In vitro Findings

#### 3.2.1. sPLA_2_-IIA Induces CF Proliferation and Collagen Synthesis

Next, we hypothesized that sPLA_2_-IIA might act as a cytokine-like modulator of cardiac damage in EAM. To test this possibility, we focused on CFs because of its key role during the pathological remodelling of the heart and we examined whether sPLA_2_-IIA could induce the transition of quiescent fibroblasts towards synthetic and proliferative myofibroblasts.

Primary cultures of adult rat CFs were treated with different doses of sPLA_2_-IIA for 24 h. These doses were previously selected according to those detected in human plasma under pathological conditions [19]. Our results revealed that CFs proliferation was stimulated in a dose-dependent manner and reached a maximal change at 1 μg/mL, the dose which was used for all subsequent experiments. AngII (1 μM) and FCS (10 %) were used as references of proliferative agonists in CFs.

**Figure 2:**
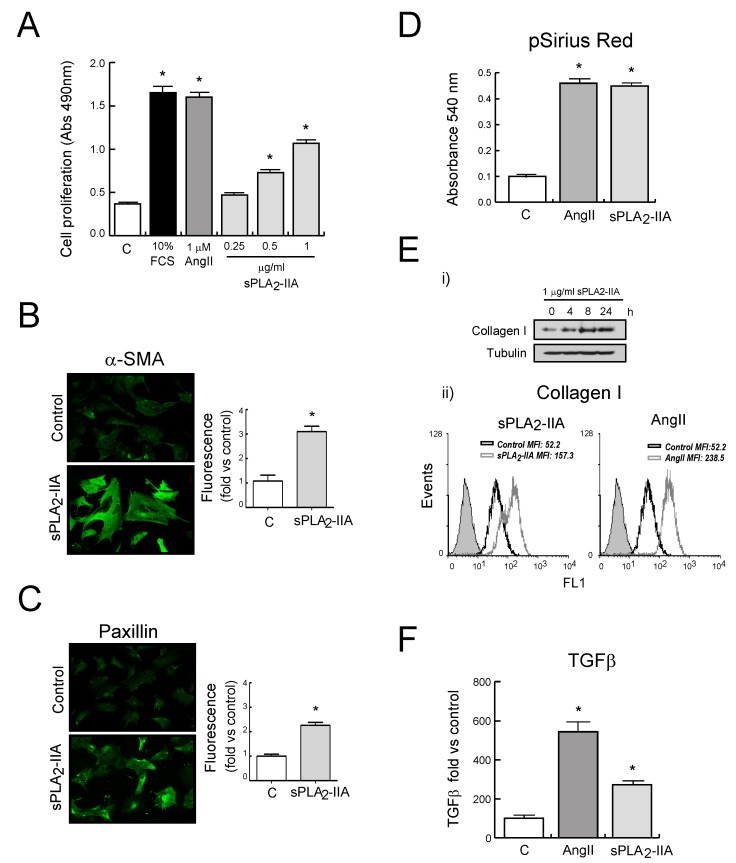
sPLA_2_-IIA modulates cardiac fibroblast (CFs) phenotype. Rat CFs were treated with the indicated stimuli. (**A**) After 24 h of incubation, cell proliferation was investigated and expressed as mean ± SD. After 24 h of incubation, αSMA (**B**) and paxillin (**C**) expression were examined by fluorescence microscopy. Representative images of resting and sPLA_2_-IIA-stimulated cells (objective lens 20×, magnification 200×) and quantitative analysis. (**D**) After 72 h of incubation, total collagen accumulation was stained with picro-Sirius red and quantified by spectrophotometric analysis. (**E**) Collagen I protein expression: (**i**) Western blot of CF stimulated at the indicated times, and (**ii**) flow cytometry histograms of CF upon 24 h stimulation (untreated-CFs (open black curves) and treated-CFs (open grey curves) were compared with isotype controls (solid grey curves)). (**F**) TGFβ expression was evaluated after 24 h of stimulation by flow cytometry analysis. The graphic represents the mean fluorescence intensity (MFI) and was shown by fold control. *p < 0.001 vs. control cells, *n* = 3.

Differentiation of CFs towards activated myofibroblasts was assessed by expression of α-SMA and paxillin, as well as by collagen production. As shown in Figure 2B,C, unstimulated CFs exhibited a low and diffuse α-SMA signal, while sPLA_2_-IIA-treated cells appeared to have high levels of α-SMA in organized filaments spanning the cell. Likewise, paxillin staining of CFs showed high-intensity fluorescence in sPLA_2_-IIA-treated cells, whereas untreated fibroblasts showed diffuse low-intensity staining. These increased expression levels of α-SMA and paxillin were also confirmed by flow cytometer analysis (Figure S1). 

Quantitative analysis of collagen accumulation on CFs monolayer cultures showed an augmented collagen deposition in sPLA_2_-IIA-treated CFs (Figure 2D). These results were in accordance with the time-dependent enhanced collagen I protein expression observed in CFs upon sPLA_2_-IIA stimulation (Figure 2Ei). Flow cytometry data also revealed increased levels of collagen I in CFs stimulated for 24 h with sPLA_2_-IIA (1 μg/mL) or AngII (1 μM) (Figure 2Eii). In addition, the expression levels of the profibrotic cytokine transforming growth factor-β (TGFβ), measured by flow cytometric analysis, were increased in CFs treated with sPLA_2_-IIA (Figure 2F).

#### 3.2.2. sPLA_2_-IIA Induces Oxidative Stress in Cardiac Fibroblast

**Figure 3:**
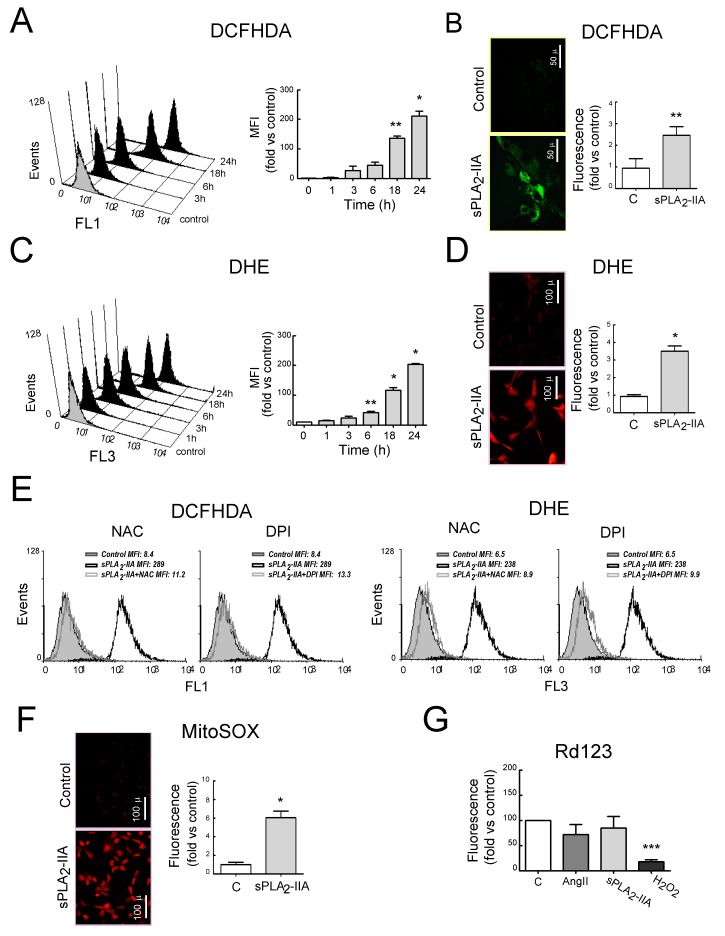
sPLA_2_-IIA induces oxidative stress in cardiac fibroblast (CFs). CFs stained with (**A**) DCFHDA, or (**C**) DHE were untreated (solid grey curves) or 1 μg/mL of sPLA_2_-IIA-treated (solid black curves) for different times. Representative histogram and mean fluorescence intensity (MFI) quantification expressed by fold of control. Resting and 24 h sPLA_2_-IIA-stimulated cells stained with (**B**) DCFHDA or (**D**) DHE, were examined by fluorescence microscopy. Representative microphotographs and quantification. (**E**) CFs pretreated with the indicated inhibitor, and stimulated with 1 μg/mL of sPLA2-IIA for 24 h, were stained with DCHFDA or DHE. Representative histograms of flow cytometry analysis, *n* = 3. sPLA_2_-IIA-treated (open black curves) and inhibitor+sPLA_2_-IIA-treated (open grey curves) CFs were compared with untreated (solid grey curves) cells. CFs stimulated with the indicated agonist for 24 h were stained with (**F**) MitoSox – representative microphotographs and quantitative analysis or (**G**) rhodamine 123—flow cytometry analysis, expressed as mean fluorescence intensity (MFI). Bar graphs represent the mean ± SD; *n* = 3. Objective lens 20×, magnification 200×; bars = 100 μm. * *p* < 0.001, ** *p* < 0.01 and *** *p* < 0.5 vs. control cells.

To determine whether sPLA_2_-IIA induces oxidative stress, which has been reported to be involved in the progression of EAM to DCM, we analyzed the intracellular ROS levels in CF exposed to sPLA_2_-IIA (1 μg/mL) at different times. As shown in Figure 3A,C, sPLA_2_-IIA enhanced intracellular ROS production on CFs in a time-dependent manner (*p* < 0.001). Representative microphotographs with fluorescence microscopy show the enhancement of H_2_O_2_ and O_2_^−^ production (Figure 3B,D). Pretreatment with either antioxidant NAC or with the flavoprotein inhibitor DPI normalised ROS values (Figure 3E).

Mitochondrial ROS production, measured by MitoSOX fluorescence using fluorescence microscopy, was also induced upon sPLA_2_-IIA stimulation (Figure 3F and Figure S2C). However, sPLA_2_-IIA treatment for up to 24 h did not induce a substantial decline in Rd123 fluorescence (Figure 3G), indicating that mitochondrial membrane potential was unaffected. A similar effect was observed in response to the reference AngII (1 μM), whereas, H_2_O_2_ (500 µM) exposure expectedly triggered a dramatic decrease in CFs Rd123 staining compared with untreated control cells (*p* < 0.05).

#### 3.2.3. sPLA_2_-IIA-Activated Intracellular Signaling Cascades in Cardiac Fibroblast Requires EGFR Transactivation and ProHB-EGF Shedding

**Figure 4:**
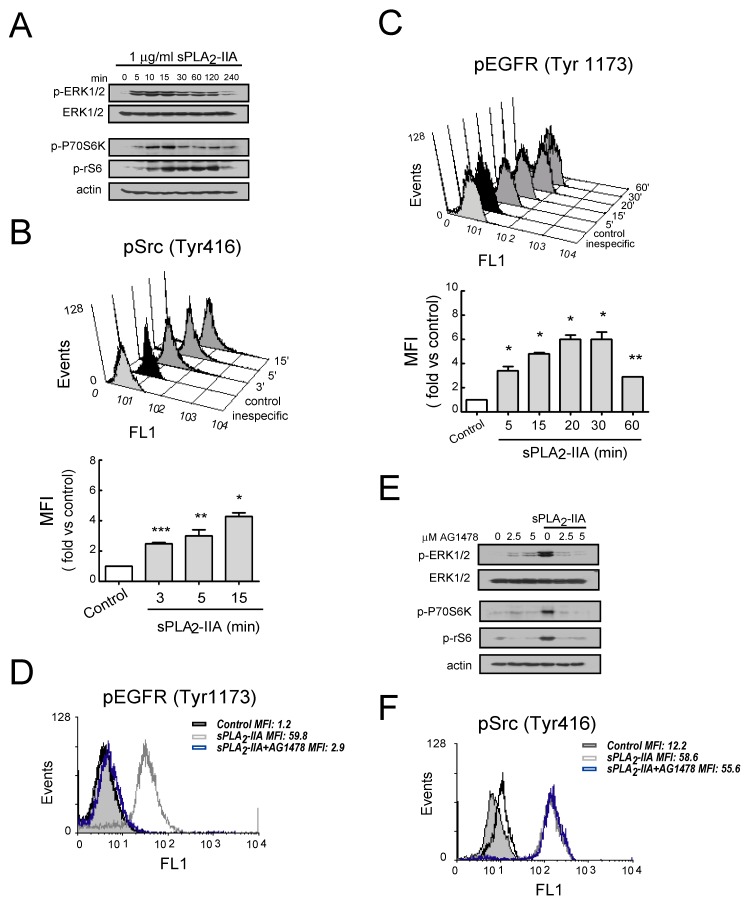
sPLA_2_-IIA-activated intracellular signaling cascades in cardiac fibroblast (CFs) requires EGFR transactivation Cells were stimulated with 1 μg/mL of sPLA_2_-IIA for indicated times: using phospho-specific antibodies, (**A**) ERK, Akt, P70S6 and S6 phosphorylation were determined by immunoblotting, while (**B**) Src and (**C**) EGFR phosphorylation was examined by flow cytometry. Untreated (solid black curves) and sPLA_2_-IIA-treated (solid dark grey curves) CFs were compared with isotype controls (solid light grey curves). (**D–F**) Cells were pretreated with AG1478 before stimulation with sPLA_2_-IIA. (**D**) After 15 min, phospho-EGFR was studied by flow cytometry. (**E**) After 15 min, ERK, Akt, P70S6 and S6 phosphorylation were determined by immunoblotting, while (**F**) Src phosphorylation was studied by flow cytometry. Untreated (open black curves), sPLA_2_-IIA-treated (open grey curves), AG1478+sPLA_2_-IIA-treated (open blue curves) CFs were compared with isotype controls (solid light grey curves). * *p* < 0.001, ** *p* < 0.01 and *** *p* < 0.5 vs. control cells; mean ± SD; *n* = 3.

Subsequently, we explore some of the signal transduction molecules involved in sPLA_2_-IIA-mediated phenotypic and functional changes in CF. As shown in Figure 4A,B, CFs stimulated with 1 μg/mL of sPLA_2_-IIA exhibited a rapid, transient and time-dependent pattern of phosphorylation in Src, ERK1/2, P70S6K and rS6 proteins.

Next, considering that EGFR and its ligands serve as a switchboard for the regulation of multiple cellular processes, we investigated whether sPLA_2_-IIA affected EGFR activity on CFs. As shown in Figure 4C, flow cytometry analysis revealed that EGFR phosphorylation at Tyr1176 increased after the addition of sPLA_2_-IIA and was suppressed by pretreatment with the selective inhibitor of EGFR tyrosine phosphorylation AG1478 (2.5 μM) (Figure 4D). sPLA_2_-IIA-induced phosphorylation of ERK1/2, P70S6K and rS6 was also inhibited by the pretreatment with AG1478 (Figure 4E), whereas this inhibitor had no effect on sPLA_2_-IIA-induced phosphorylation of Src (Figure 4F). Moreover, sPLA_2_-IIA-induced ROS production was also inhibited by the pretreatment with AG1478 (Figure S2).

**Figure 5:**
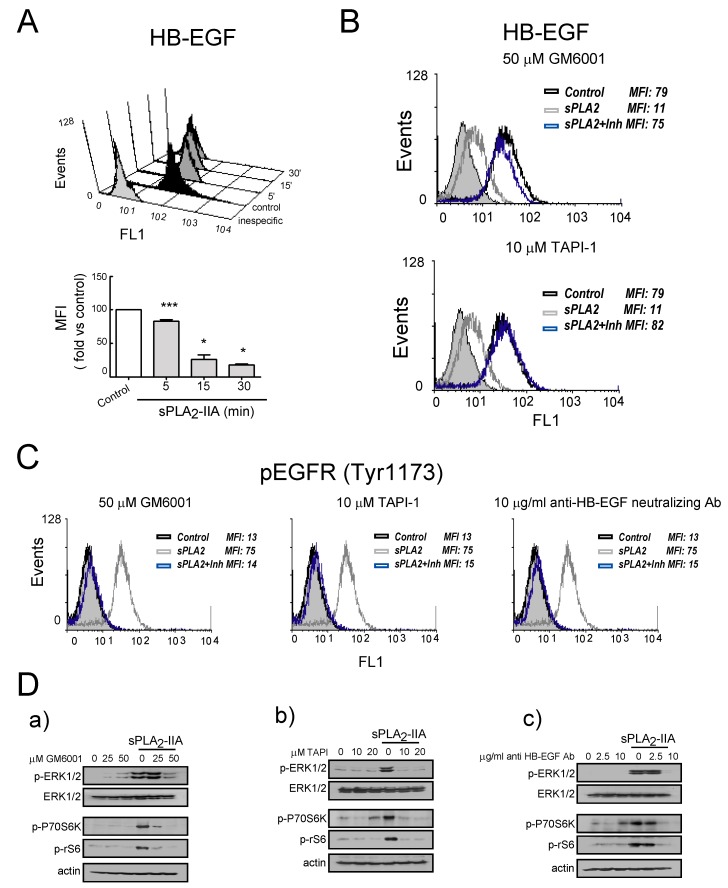
sPLA_2_-IIA-activated intracellular signaling cascades in cardiac fibroblasts (CFs) require HB-EGF shedding. (**A**) Cells were stimulated with 1 μg/mL of sPLA_2_-IIA for the indicated times, and HB-EGF expression was determined by flow cytometry: untreated (solid black curves) and sPLA_2_-IIA-treated (solid dark grey curves) CFs were compared with isotype controls (solid light grey curves). * *p* < 0.001, ** *p* < 0.01, and *** *p* < 0.5 vs. control cells; mean ± SD CFs were pretreated with the indicated inhibitors and then incubated with 1 μg/mL of sPLA_2_-IIA: after 15 min, HB-EGF (**B**) and phospho-EGFR (**C**) expression were analysed by flow cytometry: untreated cells (open black curves) were compared with sPLA_2_-IIA-treated cells in the absence (open dark grey curves) or presence of the indicated inhibitors (open blue curves). Solid light grey curves show isotype controls. (**D**) After 15 min, ERK, Akt, P70S6 and S6 phosphorylation were determined by immunoblotting. *n* = 3.

After that, we determined whether EGFR transactivation induced by sPLA_2_-IIA involves shedding of heparin-binding epidermal growth factor (HB-EGF). As illustrated in Figure 5A, flow cytometry analysis of CFs confirmed that the cell membrane-associated pool of HB-EGF decreased in response to sPLA_2_-IIA in a time-dependent manner. CFs pre-treatment for 30 min with GM6001 (50 μM), a broad-spectrum matrix metalloproteinase inhibitor, resulted in complete inhibition of HB-EGF shedding (Figure 5B). A similar pattern was observed in the presence of TAPI-1 (10 μM), an inhibitor of proteases containing a disintegrin and metalloproteinase (ADAM) domain/tumor necrosis factor-α-converting enzyme (TACE). These results suggest that sPLA_2_-IIA induces the shedding of pro-HB-EGF on CFs through an ADAMs-mediated mechanism. Inhibition of Src kinase with PP2 also prevented sPLA_2_-IIA-induced HB-EGF release, while pre-treatment with the MEK inhibitor and suppressor of the MEK-ERK signaling PD098059 had no effect (data not shown). Likewise, as shown in Figure 5C,D, the phosphorylation of EGFR and/or the signaling mediators ERK, P70S6K and rS6 was abrogated by CFs pretreatment with GM6001 (50 μM) or TAPI-1 (10 μM), as well as with the anti-HB-EGF- neutralizing antibody (10 μg/mL). This further confirms the role of pro-HB-EGF ectodomain in sPLA_2_-II-induced EGFR transactivation and signaling. None of the above-mentioned inhibitors affected phosphorylation of Src kinase (data not shown).

Finally, we also observed that sPLA_2_-IIA-induced ROS increase in CFs was abrogated through the use of the inhibitors GM6001 and TAPI-1, as well as the anti-HB-EGF neutralizing antibody (Figure S3) On resting cells, the presence of these inhibitors or the neutralizing antibody did not affect constitutive pro-HB-EGF cellular levels (data not shown).

#### 3.2.4. sPLA_2_-IIA-Induced Transactivation of EGFR Regulates Collagen Production and Maturation in CFs

**Figure 6:**
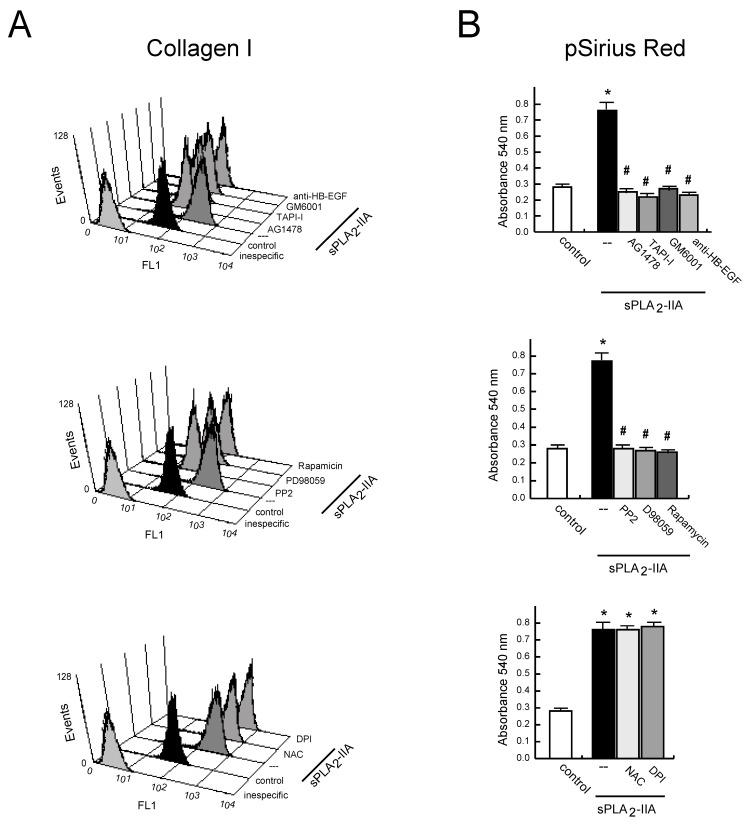
sPLA_2_-IIA induces collagen production in cardiac fibroblast (CFs). CFs pretreated without/with the indicated inhibitors, were stimulated with 1 μg/mL of sPLA_2_-IIA. (**A**) After 24 h, collagen I protein expression was evaluated on cells by flow cytometry. Untreated (solid black curves) and sPLA_2_-IIA-treated (solid dark grey curves) CFs were compared with isotype controls (solid light grey curves). Representative histograms are shown, n = 3. (**B**) After 72 h, total collagen accumulation on CFs cultures was stained with picro-Sirius red and quantified by spectrophotometric analysis. Bar graphs represent the mean ± SD; n = 3. * *p* < 0.001 vs. control cells, # *p* < 0.001 vs. sPLA2-IIA-treated cells.

**Figure 7:**
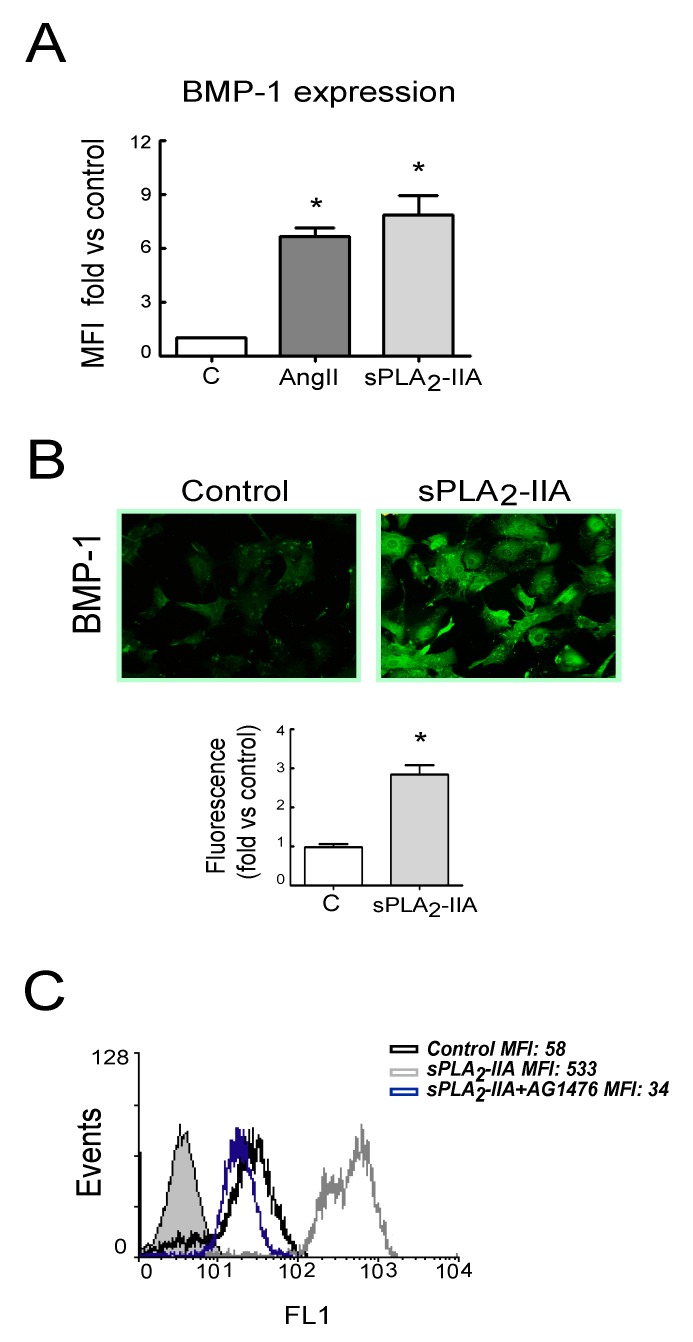
sPLA_2_-IIA induces BMP-1 expression in cardiac fibroblast (CFs) via EGFR transactivation. CFs were treated with 1 μg/mL of sPLA2-IIA or 1 μM AngII for 24 h and BMP-1 protein expression was evaluated by (**A**) flow cytometry analysis. The graphic represents the mean fluorescence intensity (MFI) and was shown by fold control. (**B**) Representative microphotographs of resting and 24 h sPLA_2_-IIA-stimulated cells examined by fluorescence microscopy (objective lens 20×, magnification 200×) and quantification. (**C**) CFs, pretreated with the EGFR inhibitor AG1476, were stimulated with 1 μg/mL of sPLA_2_-IIA for 24 h. BMP-1 expression was analyzed by flow cytometry: untreated cells (open black curves) were compared with sPLA_2_-IIA-treated cells in the absence (open dark grey curves) or presence of the inhibitor (open blue curves). Solid light grey curve shows isotype control. Representative histograms are shown, *n* = 3. Bar graphs represent the mean ± SD; *n* = 3. * *p* < 0.001 vs. control cells.

**Figure 8:**
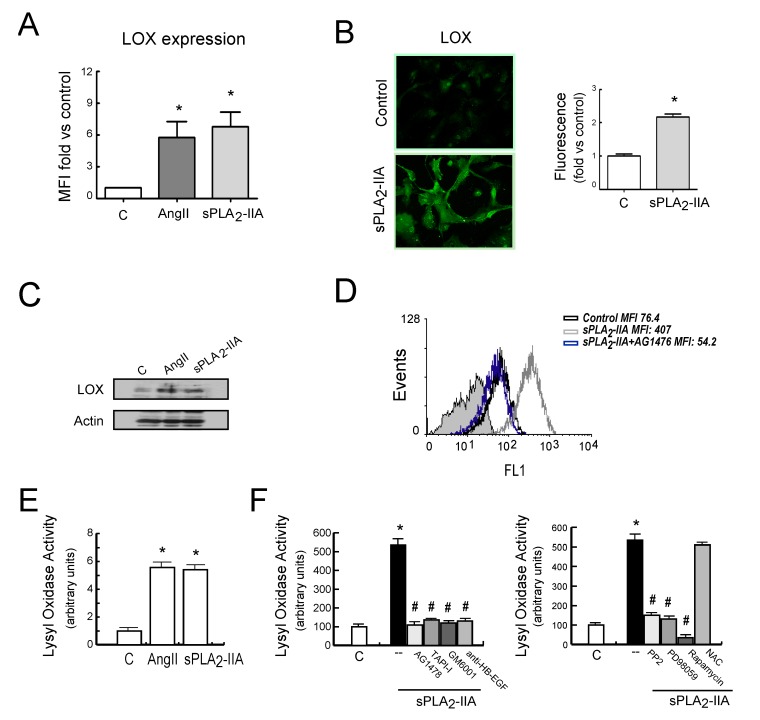
sPLA_2_-IIA induces LOX expression in cardiac fibroblast (CFs) via EGFR transactivation. CFs were treated with 1 μg/mL of sPLA_2_-IIA or 1 μM AngII for 24 h and LOX protein expression was evaluated by (**A**) flow cytometry analysis, (**B**) fluorescence microscopy and (**C**) Western blot. (**A**) The graphic represents the mean fluorescence intensity (MFI) and is shown by fold control. (B) Representative microphotographs (objective lens 20×, magnification 200×) and quantification. (**C**) Representative Western blotting in total cell lysates. (**D**) CFs, pretreated with the EGFR inhibitor AG1476, were stimulated with 1 μg/mL of sPLA_2_-IIA for 24 h. LOX expression was analysed by flow cytometry. Representative histograms are shown, n = 3. Untreated cells (open black curves) were compared with sPLA_2_-IIA-treated cells in the absence (open dark grey curves) or presence of inhibitor (open blue curves). Solid light grey curve represent isotype control. CFs were stimulated with the indicated agonists (**E**) or with 1 μg/mL of sPLA_2_-IIA in presence of the indicated inhibitors (**F**) for 24 h, and LOX activity was measured in cell culture medium. Bar graphs represent the mean ± SD; n = 3. * *p* < 0.001 vs. control cells, # *p* < 0.001 vs. sPLA_2_-IIA-treated cells.

Next, we investigated whether EGFR transactivation was involved in sPLA_2_-IIA-induced collagen production. The results showed that 24 h after sPLA_2_-IIA exposure, CFs increased collagen I expression (up to ~5-fold) measured by flow cytometry analysis (Figure 6A). The mean fluorescence intensity was 59.6 ± 8 in non-treated cells, while it was 307.3 ± 80 (*p* < 0.05) after sPLA_2_-IIA treatment. This response was completely prevented by the presence of inhibitors that block EGFR transactivation and signaling (AG1478, GM6001, TAPI-1), as well as by inhibitors of the intracellular protein kinase cascades Src–MEK/ERK–mTOR/P70S6K/rS6 (PP2, PD098059, Rapamycin). In contrast, molecules inhibiting oxidative stress (DPI, NAC) did not alter the up-regulated collagen I synthesis. We also measured collagen accumulation by a Sirius Red assay on CFs exposed to sPLA_2_-IIA for 3 days. As shown in Figure 6B, collagen deposition was significantly higher in stimulated cells compared with resting control, and the presence of the above-mentioned kinase inhibitors also abrogated collagen build-up. The antioxidants, NAC and DPI, did not affect the increased collagen expression levels triggered by sPLA_2_-IIA Next, to further investigate the relationship among sPLA_2_-IIA and factors involved in the maturation of collagen fibers, we studied the expression of BMP-1 and LOX in CFs treated with sPLA_2_-IIA for 24 h. The data indicated that sPLA_2_-IIA induces BMP-1 and LOX upregulation, reaching similar expression levels to those observed in response to the reference agonists AngII (Figure 7A and Figure 8A, respectively), supporting that sPLA_2_-IIA not only favours collagen production but also collagen cross-linking. These findings were also verified by examination of the cells under a fluorescence microscope (Figure 7B and Figure 8B, respectively). In addition, LOX up-regulation was also confirmed by Western blot analysis (Figure 8C).

As already mentioned for collagen synthesis, sPLA_2_-IIA-induced BMP-1 and LOX expression were also prevented by the EGFR inhibitor AG1478 (Figure 7C and Figure 8D, respectively). Similarly, CFs pretreatment with inhibitors that blocked EGFR transactivation, as well as with inhibitors of the intracellular protein kinase cascades such as PP2, PD098059 and Rapamycin, also abolished the induction of these proteins (Figure S4A,B,D,E). In contrast, the presence of the ROS inhibitors, NAC or DPI, did not affect their increased expression levels promoted by sPLA_2_-IIA (Figure S4C,F).

Additionally, LOX enzymatic activity was measured in the conditioned medium of CFs treated with sPLA_2_-IIA for 24 h, in the absence or presence of the above-mentioned inhibitors (Figure 8E,F). sPLA_2_-IIA increased LOX activity into the cell culture media 5-fold compared to control levels (*p* < 0.001), which was similar to that observed with 1 μM of AngII, our reference agonist. Pre-treatment with the selective protein-kinase inhibitors abolished this up-regulation. The antioxidant NAC did not affect sPLA_2_-IIA augmented LOX activity in the cell-conditioned medium.

## 4. Discussion

The involvement of sPLA_2_-IIA in chronic and acute inflammatory diseases has been well documented, but its precise role in myocardial disorders remains to be determined [14,15,16,17,18]. The present study explored molecular mechanisms regulated by the phospholipase that directly affects cardiac fibroblast phenotype and functions. Our data show that cellular infiltration and interstitial fibrosis in hearts from EAM mice were accompanied by an up-regulation of sPLA_2_-IIA protein levels. These high sPLA_2_-IIA levels were also reflected in serum. More interestingly, we observed that CFs, the predominant secretory cells producing ECM components, may be an important target of sPLA_2_-IIA. In cultures of adult rat CFs, sPLA_2_-IIA induced cell proliferation and increased expression of collagen I, TGFβ, LOX and BMP-1 protein, which are major contributing factors to the development of cardiac fibrosis.

Fibrosis results from ECM synthesis/degradation balance. LOX plays a crucial role in the maintenance of extracellular matrix stability and could participate in cardiac remodeling associated with the pathogenesis of myocardial diseases. Several studies have indicated that BMP-1 activates LOX precursor (pro-LOX) to mature active form, which is responsible for the cross-linking of collagen fibers. This allows for the formation of the mature and insoluble ECM, which is less prone to degradation [12]. LOX activity is required for normal processing of collagen, yet over-activation of LOX is associated with fibrosis. Thus, an increased expression and activity of LOX has been demonstrated in the myocardium of patients with heart failure, as well as with dilated cardiomyopathy, which correlated with increased collagen content and collagen cross-linking [27,28]. Although experimental rodent models of several cardiac disorders have enabled the assessment of the role of LOX activity in the progression of cardiac dysfunction and adverse ECM alterations, further studies are needed to identify accurately injurious stimuli, as well as cellular mechanisms responsible for the increased LOX expression and LOX-dependent damage in the heart [29,30].

In this study, in addition to the enhanced expression of structural (i.e., collagen) and matricellular (i.e., LOX, BMP-1) ECM proteins found on sPLA_2_-IIA-treated CFs, higher LOX activity was detected in the supernatants of sPLA_2_-IIA-treated CFs cultures, which was consistent with the increased collagen deposition observed in the monolayers of stimulated CFs. These findings agree with the hypothesis that sPLA_2_-IIA may act as a cardiac profibrotic factor, potentiating not only ECM production but also its cross-linking and consequently making it more difficult to degrade.

The potential role of sPLA_2_-IIA in fibrotic disorders has been suggested in the past but based mainly on indirect evidence. Mice transfected with the human *sPLA_2_-IIA* gene have increased collagen levels in atherosclerotic lesions, and spontaneously hypertensive rats treated with a specific sPLA_2_-IIA inhibitor showed a reduction in cardiac fibrosis during the development of hypertension [20,21]. However, to our knowledge, these data are the first to directly demonstrate the regulation of structural and matricellular ECM proteins in adult CFs by the inflammatory protein sPLA_2_-IIA, thus suggesting a pathogenic role in inflammatory cardiac disorders. However, sPLA_2_-IIA might not be the only one in the family, since recent evidence in a model of myocardial ischemia has pointed out that other isoforms, sPLA_2_-IB and sPLA_2_-IIE and its receptor PLA_2_R1, might mediate collagen-dependent biological effects in the infarcted myocardium [31].

Regarding the mechanism involved in sPLA_2_-IIA pro-fibrotic activity, another significant finding of the present study was that this phospholipase enhanced phosphorylation/activation of signal transduction molecules such as MEK/ERK–mTOR/P70S6K/rS6 and production of ROS through transactivation of EGFR.

The EGFR family and its ligands serve as a switchboard for the regulation of multiple cellular processes. EGFR—via transactivation—has the potential to mediate signaling of non-EGFR ligands and thereby serve as a heterologous transducer of cellular signaling. EGFR transactivation in cardiac cells has been observed following stimulation with several vasoactive substances and well-known inducers of cardiomyocyte hypertrophy such as AngII, phenylephrine, endothelin-1 and aldosterone [32,33,34,35]. In CFs, β-adrenergic receptor-dependent changes in cytokine expression are also predominantly mediated through an EGFR-sensitive manner [36]. Moreover, it has recently been shown that EGFR transactivation is also involved in ROS generation and cell apoptosis induced by high concentrations of glucose in rat cardiomyocytes, thus pointing to its participation in diabetes-induced cardiac injury [37]. Here, our data reinforce the role of sPLA_2_-IIA in inflammatory heart disorders where EGFR might serve as a signaling hub, engaging in cross-talk with multiple pathways.

To determine some of the mechanisms that link sPLA_2_-IIA signaling to EGFR transactivation on CFs, studies using a neutralizing antibody for HB-EGF, the metalloproteinase inhibitor Batimastat/GM6001, and the TACE inhibitor TAPI-1, have suggested that processing of the membrane-anchored pro-HB-EGF via matrix metalloproteinases activity (most likely TACE) is required for sPLA_2_-IIA-induced tyrosine phosphorylation of the EGFR. These data are in agreement with a series of studies, suggesting that the ADAM/MMP protease-mediated shedding of membrane-tethered ligands is a key step to activating EGFR and downstream signaling pathways. HB-EGF is known to induce cell activation in various cell types, including cardiac cells via transactivation of EGFR. In CFs and cardiomyocytes, it has been demonstrated that cellular signaling induced by inflammatory agonists and proteases, closely related to cardiac remodeling, is dependent upon HB-EGF shedding and subsequent EGFR activation [33,38,39]. Therefore, further approaches, such as knockdown EGFR or HB-EGF expression, might be considered to be future strategies for validating the results obtained with pharmacological interventions. 

Upon ligand binding, EGFR transactivation elicits downstream activation of several signalling cascades involving classic second messengers, protein kinases, non-receptors tyrosine kinases and ROS, among others [40]. In our study, we found that the selective inhibitor of the Src tyrosin kinase, PP2, blocked sPLA_2_-IIA-induced HB-EGF shedding and EGFR phosphorylation, which indicates that Src phosphorylation is an upstream signal required for sPLA2-IIA-induced CF activation. However, we observed that MEK/ERK-mTOR/P70S6K/rS6 are essential intracellular signalling molecules downstream of EGFR because CFs treated with selective inhibitors of these pathways did not modify the HB–EGF membrane levels nor blunt EGFR transactivation. sPLA_2_-IIA-induced ROS generation was also downstream of EGFR phosphorylation and required its transactivation-dependent MMP shedding of EGF-like ligands.

Moreover, we observed that on CFs stimulated with sPLA_2_-IIA, the increased synthesis of LOX, BMP-1 and collagen I proteins requires activation of EGFR after the shedding of HB-EGF from the cell surface, based on the complete abrogation of these changes by a neutralizing antibody specific for HB–EGF or metalloproteinase specific inhibitors. Additionally, active MEK/ERK–mTOR/P70S6K/rS6 signalling in CFs was crucial to orchestrate the profibrotic effects of sPLA_2_-IIA, since pharmacologic inhibition of these pathways significantly decreased the production of ECM compounds in response to sPLA_2_-IIA. However, although several *in vitro* studies have demonstrated that oxidative stress is involved in the profibrotic actions of agonist such as TGF-β1 or leptin, our data indicate that ROS production was not necessary for the enhanced matrix protein components production triggered by sPLA_2_-IIA on CFs [35,41].

Moreover, the data show that sPLA_2_-IIA not only stimulates ECM production but also favours the transformation of CFs into cardiomyofibroblasts with more capacity for ECM production and proliferation, a normal process that occurs in response to myocardial injury [12,13].

The present study reveals some of the molecular mechanism involved in the phenotypic changes induced by sPLA_2_-IIA in adult CFs, although there are still many missing steps and uncertainties that need to be studied in depth, e.g., membrane molecular effectors acting upstream HB–EGF shedding and connecting signalling events and the biological effect of sPLA_2_-IIA. Moreover, based on existing findings, a direct sPLA_2_-IIA-integrin interaction is an interesting hypothesis to address CFs in future studies [42].

In summary, in this study, we highlight the complexity of the molecular mechanisms involved in myocardial fibrosis, providing data to understand the role of sPLA_2_-IIA in pathological conditions affecting heart function. According to the pivotal role of CFs in cardiac disease and remodelling, we believe that these observations will raise significant clinical interest.

## Supplementary Materials

The following are available online at https://www.mdpi.com/2073-4409/9/2/396/s1, Figure S1: sPLA_2_-IIA modulates cardiac fibroblasts phenotype, Figure S2: Effect of the EGFR inhibitor AG1478 on sPLA_2_-IIA-induced ROS accumulation in cardiac fibroblasts, Figure S3: Effect of inhibitors that block EGFR transactivation on sPLA_2_-IIA-induced ROS increase in cardiac fibroblasts, Figure S4: Effect of selected inhibitors on sPLA_2_-IIA-induced BMP-1 and LOX expression in cardiac fibroblasts.
